# Comparison of midazolam and dexmedetomidine for pain relief during and after hysterosalpingography in women with infertility

**DOI:** 10.25122/jml-2019-0013

**Published:** 2019

**Authors:** Fatemeh Safi, Leila Rabiee, Maryam Shokrpour, Alireza Kamali

**Affiliations:** 1. Department of Radiology, Arak University of Medical Sciences, Arak, Iran; 2. Department of Gynecology, Arak University of Medical Sciences, Arak, Iran; 3. Department of Anesthesiology and Critical Care, Arak University of Medical Sciences, Arak, Iran

**Keywords:** Pain, dexmedetomidine, hysterosalpingography, midazolam

## Abstract

Patients feel uncomfortable with cervical manipulation, uterine distension and stimulation of peritoneum during hysterosalpingography (HSG) and experience lower abdominal pain during and after the procedure. Pain during the procedure has a negative effect on the adaptation of patients to treatment and physicians are trying to overcome this unpleasant situation. Therefore, the aim of this study was to compare the effect of midazolam and dexmedetomidine on reducing pain and spasm of fallopian tubes during and after HSG procedure in women with infertility. In a double-blind randomized controlled trial, 102 patients were randomly divided into two groups, midazolam and dexmedetomidine. The pain was recorded during injection and immediately after injection and 30 minutes after HSG, and then the complications of injection were recorded. Finally, the data were analyzed using SPSS version 20. Based on the results presented herein, no significant difference was found between the two groups in terms of vasovagal reaction, spasticity of the tube and the side of the spastic tube and uterine cavity anomalies (p <0.05). However, the pain showed a significant difference between the two groups during the injection, immediately or at 30 minutes after the procedure (p = 0.0001). The pain in the midazolam group was less than that of dexmedetomidine. Furthermore, there was no significant difference between the two groups regarding spasticity (p <0.05). There is a benefit in terms of pain reduction with the use of dexmedetomidine when comparing with midazolam injection. However, dexmedetomidine does not cause side effects in patients and can be used to reduce pain during injection.

## Introduction

Infertility is one of the most common problems in women’s medicine. This complication involves a heterogeneous group of patients and infertility-related assessments require special attention to determine the cause of infertility. Hysterosalpingography (HSG) has become a commonly performed diagnostic method for assessing the inside of the uterine cavity and fallopian tubes [1]. Due to the reliability, availability, simplicity, and cost-effectiveness of this method, it is very difficult to replace the other diagnostic method with HSG [2]. In 2004, the *National Institute* for Health and Care Excellence (*NICE*) recommended HSG guidelines as a useful approach for screening for *blocked fallopian tubes* [3]*.* Although this procedure is non-invasive, which does not require cervical dilatation or anesthesia, however, the patients expressed discomfort because of manipulation of the cervix (especially by the Tenaculum), uterine distention and stimulation of peritoneum, where they experienced lower abdominal pain during and after the procedure. Pain during the procedure has a negative impact on the adaptation of patients to the treatment and physicians are trying to overcome this unpleasant situation [1]. Many studies have evaluated the effects of various types of drug interventions, including *non-opioid analgesics* versus placebo [4], and opioid analgesics versus *non-opioid analgesics* [5]*,* as well as local anesthetics and placebo [6-8]. However, it seems that there is still no consensus about how to produce analgesia and the time to do it. To the best of our knowledge, there has been no study on the effect of medication on a *spasm* of the *fallopian tubes* during HSG, and most studies have been conducted to reduce the pain of these patients during the procedure. The effect of dexmedetomidine and midazolam on pain and spasm of fallopian tubes has not been studied so far. Therefore, the main aim of this study was to determine the effect of dexmedetomidine and midazolam on pain and spasm of fallopian tubes during and after HSG in women with infertility.

## Material and Methods

This *double-blind randomized controlled trial* was performed on patients undergoing HSG, referring to the radiology clinic in Arak, Iran. In this study, 102 patients were randomly divided into two groups, midazolam and dexmedetomidine, using *hypercube sampling.*

Inclusion criteria included women with infertility, aged 20-45 years, having informed consent, contraindication for the use of dexmedetomidine, contraindication of the use of midazolam, no known uterine malignancy, lack of vaginal bleeding, no history of cervical surgery, no PID, absence of painkiller use before HSG and fallopian tube obstruction. Exclusion criteria included HSG duration greater than 45 minutes and presence of unstable hemodynamic symptoms during HSG. The method was explained to the selected patients and they were consciously entered into the study after obtaining written consent. All women were placed in the follicular phase of the *menstrual cycle* to participate in this study. The patient was placed in a supine position after being prepared on the bed and peripheral venous catheter 20 was placed into a peripheral vein for all of them. In group 1, 1μg/kg of dexmedetomidine (intravenous *bolus injection;* 3 ml) was injected 3-4 minutes before the procedure; subjects in group 1 then received an *infusion* of 0.5mg/kg *dexmedetomidine*. The second group received *infusion* doses of midazolam, 0.4 mg/kg (volume: 3 ml) 3-4 minutes before the procedure. Normal saline (3 ml; placebo group) was infused into patients in the second group. After *achieving* target *sedation* of grade *3* on the *Ramsay scale*, the process was started and the patient was placed in a lithotomy position. Afterward, a *sterile speculum* was placed inside the vagina. After the cervical vision, a betadine solution was used for *scrubbing of* area. The anterior or posterior edges of the cervix were then taken by the tenaculum and the cannula of the *hysterosalpingogram* was entered in the cervical canal. The speculum was then removed and 5 cc contrast agent was injected. At this time, the patient was asked to determine their pain intensity using the visual analog scale. The presence or absence of spasm of fallopian tubes was determined at this stage. In the absence of contrast in the uterine tubes, spasticity was detected after the first and second injection. However, if the contrast agent did not pass through the uterine tubes, after repeated injections and patient relaxation, fallopian tube obstruction was revealed and the patient was excluded from the study.

At the end of the procedure, all instruments were removed, and the patients were monitored for half an hour in the clinic. The patients were also asked about pain after HSG thirty minutes after the completion of work. In addition, patients were also questioned about vasovagal symptoms including nausea, vomiting, sweating, weakness, hypotension, and bradycardia 30 minutes after the procedure. Data were then analyzed by SPSS software v 20.

## Results

In the midazolam group, 57 patients were examined, of which six were excluded from the study due to tube obstruction, and 55 patients in the dexmedetomidine group were examined, of which four were also excluded. One patient was excluded from the study due to hemodynamic impairment and pressure drop and the other three were excluded because of the tube obstruction. Moreover, the minimum age was recorded as 19 years and the maximum age was 43 years. The mean age of patients was also determined to be 29.15 ± 5.24 years. Duration of infertility in the dexmedetomidine group was 25.64 ± 19.99 months and this period was also found to be 26.09 ± 23.66 months for midazolam group, where no significant difference was found between the two groups (p = 0.916). According to the results, there was no significant difference between the two groups in terms of age (p = 0.98). In addition, there was no significant difference between the two groups regarding parity, dysmenorrhea, history of dyspareunia, history of HSG and history of endometrial biopsy (p <0.05). Furthermore, no significant difference was found between the two groups in the duration of HSG (p = 0.878). Based on the data presented herein, two groups did not show a significant difference in terms of vasovagal reaction and uterine cavity anomalies (p <0.05). Additionally, there was no significant difference between the two groups regarding spasticity of the tube (p <0.05), (Tables 1 and 2).

**Table 1:** Comparison of complications during HSG in the two groups of dexmedetomidine and midazolam

Group	Variable	Dexmedetomidine Number(%)	Midazolam Number(%)	p-value
Vasovagal reaction	Yes	(0)0	(0)0	>0/05
	No	(100) 51	(100)51	
Uterine cavity anomalies	No	(98/03)50	(98/03)50	0/368
	*polyp*	(0)0	(1/96)1	
	Submucosal Myoma	(1/96)1	(0)0	
	Septum	(0)0	(0)0	
	Endometrium Adhesion	(0)0	(0)0	
	Other	(0)0	(0)0	

**Table 2:** Comparison of spasticity in two groups of dexmedetomidine and midazolam

Group	Variable	Dexmedetomidine Number(%)	Midazolam Number(%)	p-value
Tube spasm	Yes	(11/76)6	(11/76)6	0/620
	No	(88/23)45	(88/23)45	
spasm Side	Right	(3/92)2	(3/92)2	>0/ 05
	Left	(5/88)3	(5/88)3	
	Bilateral	(1/96)1	(1/96)1	

**Figure 1:**
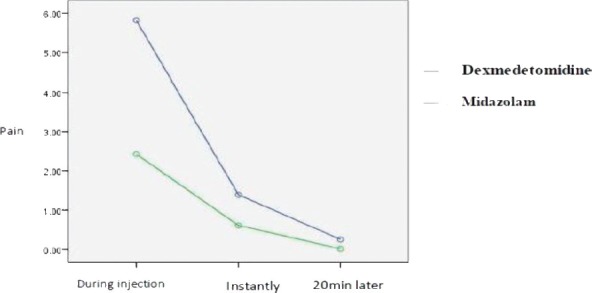
Comparison of pain at different times in the two groups of dexmedetomidine and midazolam

As indicated in Table 3, pain at all times showed a significant difference between the two groups (p <0.05), where the pain in the midazolam group was less than that of the dexmedetomidine group. (Table 3; figure 1).

**Table 3:** Comparison of mean and standard deviation of pain in the two groups

Group	Variable	Dexmedetomidine Number(%)	Midazolam Number(%)	p-value
Tube spasm	Yes	(11/76)6	(11/76)6	0/620
	No	(88/23)45	(88/23)45	
Spasm location	Right	(3/92)2	(3/92)2	>0/05
	Left	(5/88)3	(5/88)3	
	Bilateral	(1/96)1	(1/96)1	

In addition, no significant difference was found between the two groups regarding the number of injection (p = 0.747).

## Discussion

Infertility is a complex situation that affects people’s lives in many ways [9]. Infertility-related studies in women require special attention in obtaining the cause of this problem [10]. Performing HSG is one of the most important diagnostic methods for assessing the openness of the uterus and uterine tubes [11]. HSG is associated with a feeling of discomfort and leads to lower abdominal pain during and after it.

In most women, this pain has a negative effect on HSG in patients, so specialists have taken different drug interventions to reduce pain in these patients [12-14]. In this regard, the current study was aimed to evaluate the effect of two drugs, dexmedetomidine and midazolam, on pain and spasm of fallopian tubes during and after HSG in women with infertility.

The findings of this study demonstrated significant difference between the two groups regarding the duration of infertility. In addition, no significant difference was found between the two groups of patients in terms of parity, dysmenorrhea, history of dyspareunia, history of HSG, and history of endometrial biopsy. There was no significant difference between two groups in terms of vasovagal reaction and anomaly of the cavity, and no significant difference was revealed in spasticity of the tube. Based on our findings, pain at all times exhibited a significant difference between the two groups (p < 0.05). The pain in the midazolam group was lower as compared to the dexmedetomidine group. No significant difference was found between the two groups regarding the duration of HSG and the frequency of injections.

**Table 4:** Comparison of mean and standard deviation of the number of injections in the two groups of dexmedmotidine and midazolam

Group Variable	Dexmedmotidine Mean±SD	Midazolam Mean±SD	p-value
Number of injections	3/41±1/06	3/35±0/743	0/747

To the best of our knowledge, there is no similar study like our survey. Therefore, we try to compare studies that focused on reducing the pain during and after HSG.

Hassa et al., evaluated the effects of oral nonsteroidal anti-inflammatory drugs (NSAIDs) and aginal misoprostol for decreasing pain during, and 30 minutes after, HSG. They demonstrated no benefit in terms of pain reduction using oral nonsteroidal pain during, and 30 minutes after, HSG procedure, while the use of NSAIDs can relieve pain 30 minutes after HSG procedure [15]. Although the aforementioned study is different from our study, our results indicate a reduction in pain during and after HSG procedure.

Another study assessed the effect of 5% lidocaine 25 mg-prilocaine 25 mg/g cream for decreasing pain during HSG. This study showed that endocervical and exocervical topical use of the EMLA 10 minutes before HSG is capable of decreasing the pain during manipulation of the cervix with tenaculum and cannula, but had no effect on the pain during the injection of contrast [16].

Gupta et al. in 2007 compared the effect of oral naproxen with intrauterine instillation of 1% lignocaine in relieving the pain caused by HSG procedure in women with infertility. This study revealed that intrauterine application of lignocaine was not capable of showing effective levels of *pain relief during* the *HSG* as compared to oral naproxen [17].

In a study by Elson and Ridley in 2000, the effect of paracetamol as a prophylactic pain reliever for HSG in the UK was evaluated. This study reported that paracetamol is not effective in relieving pain caused by the HSG procedure [4].

In the current study, dexmedetomidine and midazolam were capable of reducing pain, but pain reduction after HSG was less in the midazolam group. Spasm did not differ between the two groups and the complications were similar in two groups.

## Conclusion

There is a favorable effect in terms of pain reduction with the application of dexmedetomidine during HSG or at 30 minutes after performing HSG as compared to the midazolam group. This is despite the fact that dexmedetomidine does not cause side effects in patients and can be used to reduce pain during injection. On the other hand, spasms were similar in two groups.

## Acknowledgment

This thesis is approved at Arak University of Medical Sciences (code IR.ARAKMU.REC.1396.). It also has a registration code at the Iranian Center for Clinical Trials (IRCT2017081520258N57). We are grateful for the material and spiritual contributions of Research Development Center of Valiasr Hospital and *Deputy of Research and Technology of Arak University of Medical Sciences.*

## Conflict of Interest

The authors confirm that there are no conflicts of interest.
